# Genetic engineering biofilms *in situ* using ultrasound‐mediated DNA delivery

**DOI:** 10.1111/1751-7915.13823

**Published:** 2021-05-16

**Authors:** Chun Kiat Ng, Samuel L. Putra, Joseph Kennerley, Robert Habgood, Ronald A. Roy, Jason L. Raymond, Ian P. Thompson, Wei E. Huang

**Affiliations:** ^1^ Department of Engineering Science University of Oxford Parks Road Oxford OX1 3PJ UK; ^2^ Oxford Suzhou Centre for Advanced Research 388 Ruoshui Road, Suzhou Industrial Park Jiangsu 215123 P.R. China

## Abstract

The ability to directly modify native and established biofilms has enormous potential in understanding microbial ecology and application of biofilm in 'real‐world' systems. However, efficient genetic transformation of established biofilms at any scale remains challenging. In this study, we applied an ultrasound‐mediated DNA delivery (UDD) technique to introduce plasmid to established non‐competent biofilms *in situ*. Two different plasmids containing genes coding for superfolder green fluorescent protein (sfGFP) and the flavin synthesis pathway were introduced into established bacterial biofilms in microfluidic flow (transformation efficiency of 3.9 ± 0.3 × 10^‐7^ cells in biofilm) and microbial fuel cells (MFCs), respectively, both employing UDD. Gene expression and functional effects of genetically modified bacterial biofilms were observed, where some cells in UDD‐treated *Pseudomonas putida* UWC1 biofilms expressed sfGFP in flow cells and UDD‐treated *Shewanella oneidensis* MR‐1 biofilms generated significantly (*P* < 0.05) greater (61%) bioelectricity production (21.9 ± 1.2 µA cm^−2^) in MFC than a wild‐type control group (~ 13.6 ± 1.6 µA cm^−2^). The effects of UDD were amplified in subsequent growth under selection pressure due to antibiotic resistance and metabolism enhancement. UDD‐induced gene transfer on biofilms grown in both microbial flow cells and MFC systems was successfully demonstrated, with working volumes of 0.16 cm^3^ and 300 cm^3^, respectively, demonstrating a significant scale‐up in operating volume. This is the first study to report on a potentially scalable direct genetic engineering method for established non‐competent biofilms, which can be exploited in enhancing their capability towards environmental, industrial and medical applications.

## Graphical abstract

In this study, we applied an ultrasound‐mediated DNA delivery (UDD) technique to introduce plasmids containing genes coding for superfolder green fluorescent protein (sfGFP) and the flavin synthesis pathway into established bacterial biofilms in microfluidic flow and microbial fuel cells (MFCs) respectively. Gene expression and functional effects of genetically modified bacterial biofilms were observed, where some cells in UDD‐treated Pseudomonas putida UWC1 biofilms expressed sfGFP in flow cells and UDD‐treated Shewanella oneidensis MR‐1 biofilms generated significantly greater (61%) bioelectricity production in MFC than a wild‐type control group. The effects of UDD were amplified in subsequent growth under selection pressure due to antibiotic resistance and metabolism enhancement. This is the first study to report on a potentially scalable direct genetic engineering method for established non‐competent biofilms, which can be exploited in enhancing their capability towards environmental, industrial and medical applications.

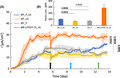

## Introduction

Microbial biofilms are one of the most widely distributed and successful modes of life on Earth, where they drive vital biogeochemical cycling processes of most elements in water, soil, sediments and subsurface environments (Stoodley *et al*., 2002). When properly deployed, biofilms can be useful and have long been exploited in bioengineered applications including the degradation of wastewater and solids applied to the filtration of potable water (Halan *et al*., 2012; Meckenstock *et al*., 2015). Compared to cells in suspension, biofilms have high cell densities, intrinsic robustness, ability to self‐renew and stable process rates, which are ideal characteristics for exploitation in bioreactors and microbial fuel cells (MFCs) to achieve biosynthesis of high‐value chemicals and biofuels (Singh *et al*., 2006; Botyanszki et al., 2015). A key feature of most biofilms is that they are composed of diverse communities, enabling them to perform functions that are difficult or impossible for individual species to achieve (Stewart and Franklin, 2008; Elias and Banin, 2012). Thus, there is a division of labour, each population undertaking their specific tasks with their metabolites acting as substrates for populations rather than metabolic cascade within individual bacterium (Hays *et al*., 2015).

The effective harnessing of microbial community functionality and robustness over operationally useful timescales remains a key challenge for the deployment of multispecies biofilms in industrial applications (Rosche *et al*., 2009). Engineers and biotechnologists are limited in what they can do when systems are performing sub‐optimally or moving towards failure. Furthermore, if poor management leads to biofilms becoming detached and dispersed (e.g. by toxic shock from a sudden influx of heavy metals into a municipal water treatment plant), it may take a long time to re‐establish a functional colony, potentially resulting in significant downtime and economic loss (Pagga *et al*., 2006). While biofilm‐based technologies, such as microbial fuel cells (MFCs), are promising and sustainable in the long term, critical bottlenecks inherent to biofilm physiology such as vulnerability to toxic shock, slow adaption to potential changing conditions, and slow biofilm growth remain to be addressed before their widespread applications in industrial settings (Rosche *et al*., 2009).

The introduction of plasmids into bacterial cells holds great promise in terms of modifying the fate and functioning of a biofilm, while addressing many of the bottlenecks associated with biofilm‐based technologies. Plasmids are physically separated from the main chromosomal DNA of the cell and can replicate independently, often endowing the cell with self‐maintaining genes which enable new functionalities and allowing the cell to deal with changing conditions (Sørensen *et al*., 2005). Bacteria have long been engineered via plasmids or other forms of nucleic acids to perform various activities, ranging from the treatment of radioactive waste (Brim *et al*., 2000), the generation of bioelectricity (Yang *et al*., 2015), the removal of heavy metals from industrial effluent (Collard *et al*., 1994) and even the visualization of the gut microbiome in bees (Leonard *et al*., 2018). However, these applications require inoculation of engineered strains into respective environments where the introduced strains may be unable to compete with native populations or existing biofilm communities and hence fail to effectively colonize and perform their intended functions (De Lorenzo, 2009). What is required is *in situ* microbiome engineering methods that enable manipulation of mature established multispecies biofilms in their native context. In our previous study, we reported on the successful applications of low frequency 42 kHz ultrasound for transferring plasmids into three different bacterial species in their planktonic states and achieved gene uptake in cells (9.8 ± 2.3 × 10^‐6^ per cell) (Song *et al*., 2007). However, there are currently no viable methods that enable introduction of desired genes into complex and niche biofilm communities (e.g. activated sludge, gut microbiome), which are predominantly comprised of non‐competent and/or non‐culturable cells.

In this study, ultrasound‐mediated DNA delivery (UDD) was developed as a non‐invasive and scalable means of genetic engineering non‐competent established biofilms to change and enhance their capabilities. We first establish the feasibility of UDD to add new functions by introducing plasmids (carrying a reporter gene) into biofilms established in microfluidic flow cells and observing the occurrence of fluorescence signals within the biofilms. Second, we demonstrate the capability of UDD to improve existing biofilm function by introducing plasmids (carrying genes encoding proteins for the synthesis of flavin – an electron shuttling molecule) into electrically active biofilms established in MFC and measuring their bioelectricity generation. Lastly, we explore the potential of scaling up the UDD technology by testing plasmid delivery into biofilms established in bioreactors of two vastly different operating volumes.

## Results

### Ultrasound‐mediated DNA delivery (UDD) in flow cell biofilms

**Fig. 1:**
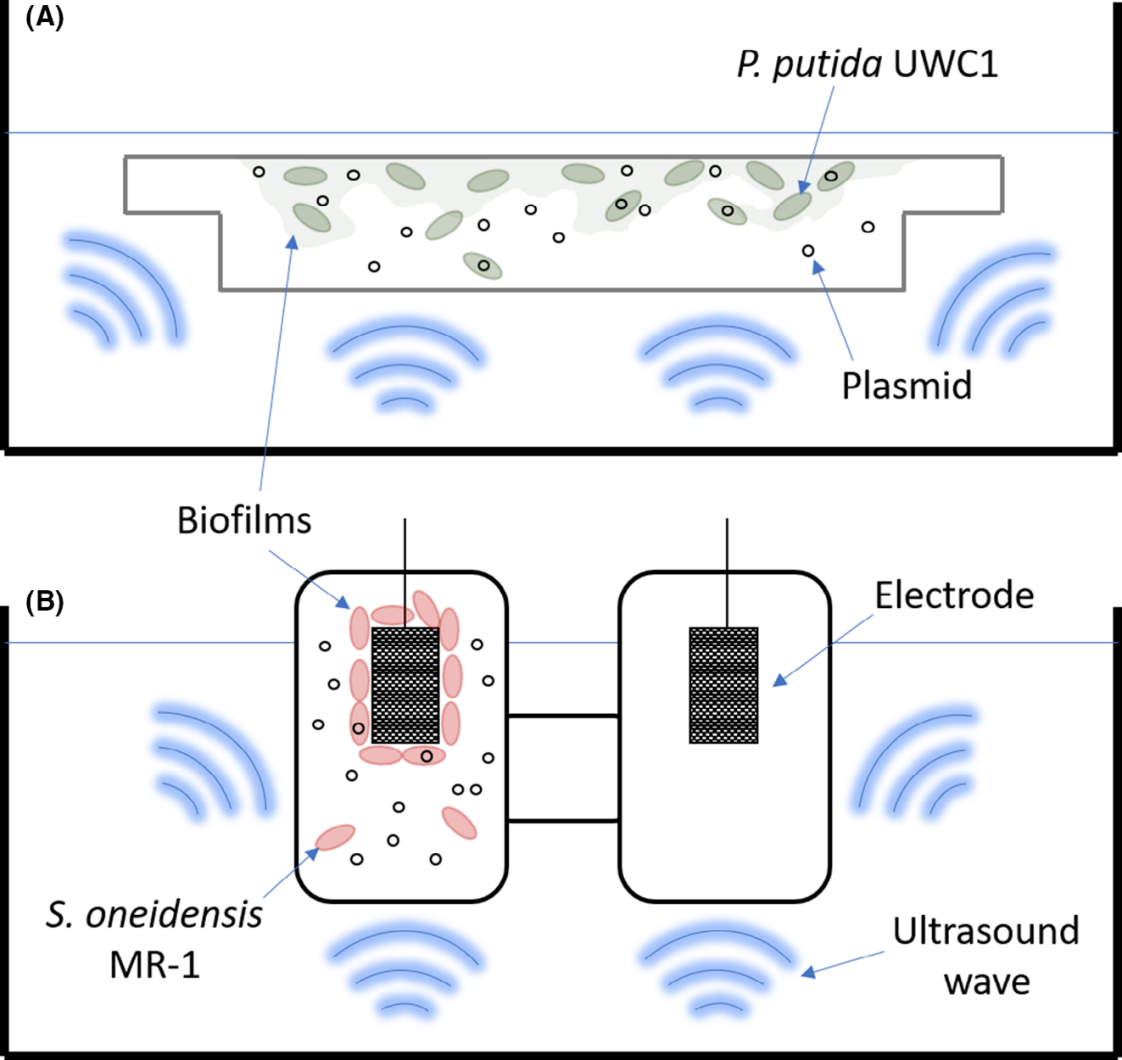
Schematic diagram of ultrasound‐based DNA delivery (UDD) into bacterial cells of mature biofilms established in (A) microfluidic flow cells and (B) microbial fuel cell (MFC). Ultrasound treatments were applied in a commercially available 42 kHz ultrasound cleaning bath. Diagram is not drawn to scale.

A UDD system consisting of samples submerged in a commercial ultrasonic water bath (see Methods section below) was set up to examine the effectiveness of ultrasound to transfer plasmids to biofilms of *Pseudomonas putida* UWC1 and *Shewanella oneidensis* MR‐1 (Fig. 1A and B). Before UDD, the *P. putida* UWC1 biofilm was established in microfluidic flow cells (Fig. 1A) and the *S. oneidensis* MR‐1 biofilm was established on an electrode surface (Fig. 1B). The frequency of ultrasound was nominally 42(± 6%) kHz continuous wave with an amplitude modulated envelope that yielded a time‐average acoustic intensity of 1.9 W cm^−2^ (see below for more details on the acoustic field properties). The ultrasound treatment time for plasmid transfer was 10 s. The operation was undertaken with the bath filled with slightly degassed (order 90% of saturation) water at laboratory temperature (order 20°C) and local atmospheric pressure (order 1 bar).

After an initial 3‐day of incubation, *P. putida* UWC1 biofilms were established in flow cells. The biofilms were treated using four different exposure conditions: plasmid addition and ultrasound treatment (+P/+U), no plasmid and only ultrasound treatment (−P/+U), only plasmid addition and no ultrasound treatment (+P/−U), and neither plasmid nor ultrasound treatment (−P/−U). The plasmid pBBR1MCS‐2_Plux_sfGFP (Table 1 and Fig. S3) contained a broad host pBBR1 backbone and the superfolder gfp gene was fused with a *lux*I promoter. The *lux*I promoter also controls *lux*I and *lux*R which form a positive feedback loop to enhance superfolder GFP (sfGFP) expression (Fig. S3).

**Table 1:** The bacterial strains and plasmids employed in this study.

Bacterial strain or plasmid	Genotype, description	References or source
Strains		
*Pseudomonas putida* UWC1	A spontaneous rifampicin‐resistant mutant of P. putida KT2440. Not naturally competent	McClure *et al*. (1989)
UWC1/sfGFP	*P. putida* UWC1: pBBR1MCS‐2_P_lux__sfGFP	This study
*Shewanella oneidensis* MR‐1 wild type (WT)	Wild‐type strain of MR‐1. Not naturally competent	Heidelberg *et al*. (2002)
MR‐1 Δbfe	Δbfe mutant of MR‐1. Loss of ability to transport the FAD into the periplasm, reduced extracellular flavins available for electron transfer	Kotloski and Gralnick (2013)
MR‐1/YYDT‐C5	*S. oneidensis* MR‐1: pYYDT‐C5	This study
*Escherichia coli* WM3064	A diaminopimelate (DAP) auxotroph due to mutation in dapA. Cannot undergo cell division without DAP	Dehio and Meyer (1997)
*Escherichia coli* C2987 NEB‐5α	A derivative of the *E. coli* DH5α. Competent cell for laboratory genetic manipulation, from New England Biolabs (U.K.)	Kostylev *et al*. (2015)
Plasmid		
pBBR1MCS‐2	Empty vector backbone with broad‐host‐range origin of replication (pBBR1) multiple cloning site with blue/white selection function, Kan^R^	Kovach *et al*. (1995)
pBBR1MCS‐2_Plux_sfGFP	Plasmid with positive‐feedback luxI and luxR system and superfolder green fluorescent protein (sfGFP), Kan^R^	This study
pTD103luxl_sfGFP	Oscillator plasmids with positive‐feedback luxI and luxR system and superfolder green fluorescent protein (sfGFP), colE1, Kan^R^	Prindle *et al*. (2012)
pYYDT‐C5	Plasmid with entire flavin biosynthesis gene cluster ribADEHC cloned from *Bacillus subtilis*, Kan^R^	Yang *et al*. (2015)

**Fig. 2:**
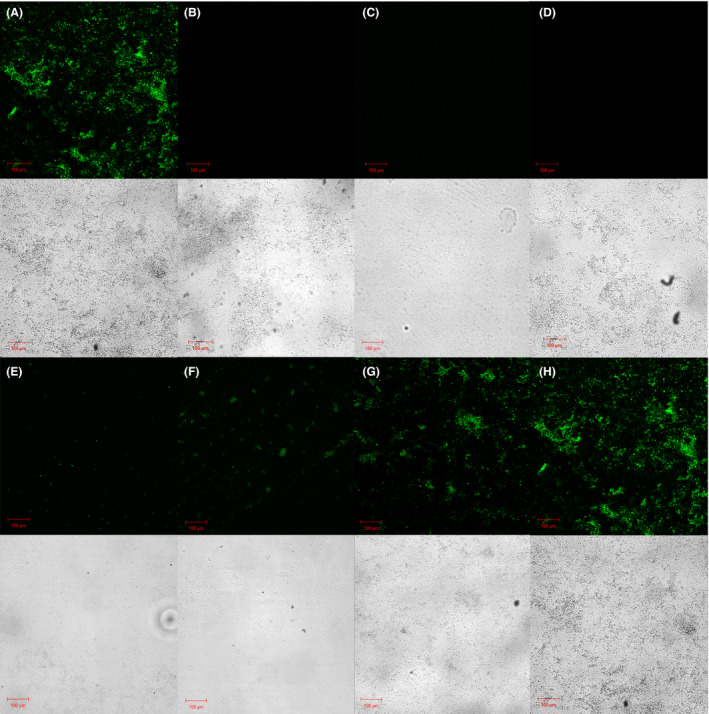
Time course of ultrasound‐mediated gene transfer into a biofilm. Green fluorescence signal (ex. 485nm, em. 510nm) and bright‐field imaging of biofilm samples after 120 h with (A) the addition of both plasmid and ultrasound treatment (+P/+U), (B) only the addition of plasmid (+P/−U), (C) only ultrasound treatment (−P/+U) and (D) no plasmid and no ultrasound treatment (−P/−U). Green fluorescence signal and bright‐field imaging for biofilm samples with both addition of plasmid and ultrasound treatment after (E) 5 h, (F) 24 h, (G) 48 h and (H) 120 h.

After treatment, all samples were subjected to an incubation period (120 h) under constant flow of growth media containing kanamycin to exert selection pressure for transformed cells over non‐transformed ones. Over a period of 120 h, some cells in *P. putida* UWC1 biofilm with plasmid and ultrasound treatment (+P/+U) expressed sfGFP (Fig. 2A), while biofilms without either ultrasound treatment (+P/‐U, Fig. 2B) or the plasmid addition (−P/+U, Fig. 2C) or neither (−P/‐U, 2d), showed no signs of sfGFP expression. The growth media outputs of the flow cell were also examined visually and only +P/+U samples showed ‘cloudiness’ indicating bacterial growth, while there were no signs of any growth in the other samples (Fig. S1). Five hours after addition of plasmid and ultrasound treatment, small green fluorescent spots of cells with sfGFP were observed across the +P/+U biofilm samples (Fig. 2E), presumably produced by transformed UWC1 cells that were expressing sfGFP from pBBR1MCS‐2_Plux_sfGFP. Clusters of green fluorescent cells with sfGFP formed after 24 h (Fig. 2F) in +P/+U biofilm samples, and the area of cells with sfGFP continued to expand over 48 (Fig. 2G) and 120 h (Fig. 2H). Ultrasound treatment may have unintentionally disrupted the structure of the biofilm and reduced its thickness. However, a layer of cells was still visible in the bright‐field image of Fig. 2E And F which consisted of the biofilm samples 5h and 24h after ultrasound treatment. The fluorescence images in Fig. 2E and F confirm that transformants were present, expressing weak but visible green fluorescent dots within the biofilm.

Effluent and biofilm samples from the flow cells in four treatments were taken and cultured in LB medium with kanamycin. Only the samples from the +P/+U treatment were able to grow, while samples from the other three controls failed to grow in the presence of kanamycin. We assumed that the abundance of transformants within biofilm is of a similar level as that in the effluent. From the effluent of the flow cell after ultrasound treatment, we measured the number of transformed bacteria to total number of bacteria and found that the percentage of transformed bacteria in the effluent was around 0.000039%, indicating the transformation efficiency was 3.9 ± 0.3 × 10^‐7^. We did not find any transformed cells in the other control groups.

The plasmids were extracted, and the sequencing of the plasmid DNA confirmed that the recovered plasmid was pBBR1MCS‐2_Plux_sfGFP. Collectively, these results demonstrate that exposure to 42 kHz ultrasound induced the transfer of plasmids into an established biofilm and provide a proof‐of‐concept that UDD can be employed to enable *in situ* genetic engineering of non‐competent established biofilms.

### Flavin‐mediated electron shuttling is the dominant mechanism of extracellular electron transfer in *Shewanella oneidensis* MR‐1

Microbial fuel cells (MFCs) produce electricity using a bacterial biofilm deposited on an electrode to oxidize organic matter (Logan *et al*., 2006). In this experiment biofilms of *S*. *oneidensis* MR‐1 wild type (WT), MR‐1 Δbfe [knockout of *bfe* gene for bacterial flavin adenine dinucleotide [FAD] exporter (Kotloski and Gralnick, 2013)] and MR‐1/YYDT‐C5 [MR‐1 with plasmid pYYDT‐C5 containing the entire flavin biosynthesis gene cluster *rib*ADEHC cloned from *Bacillus subtilis* (Liu *et al*., 2015; Yang *et al*., 2015)] were established in the microbial fuel cell (MFC) system.

**Fig. 3:**
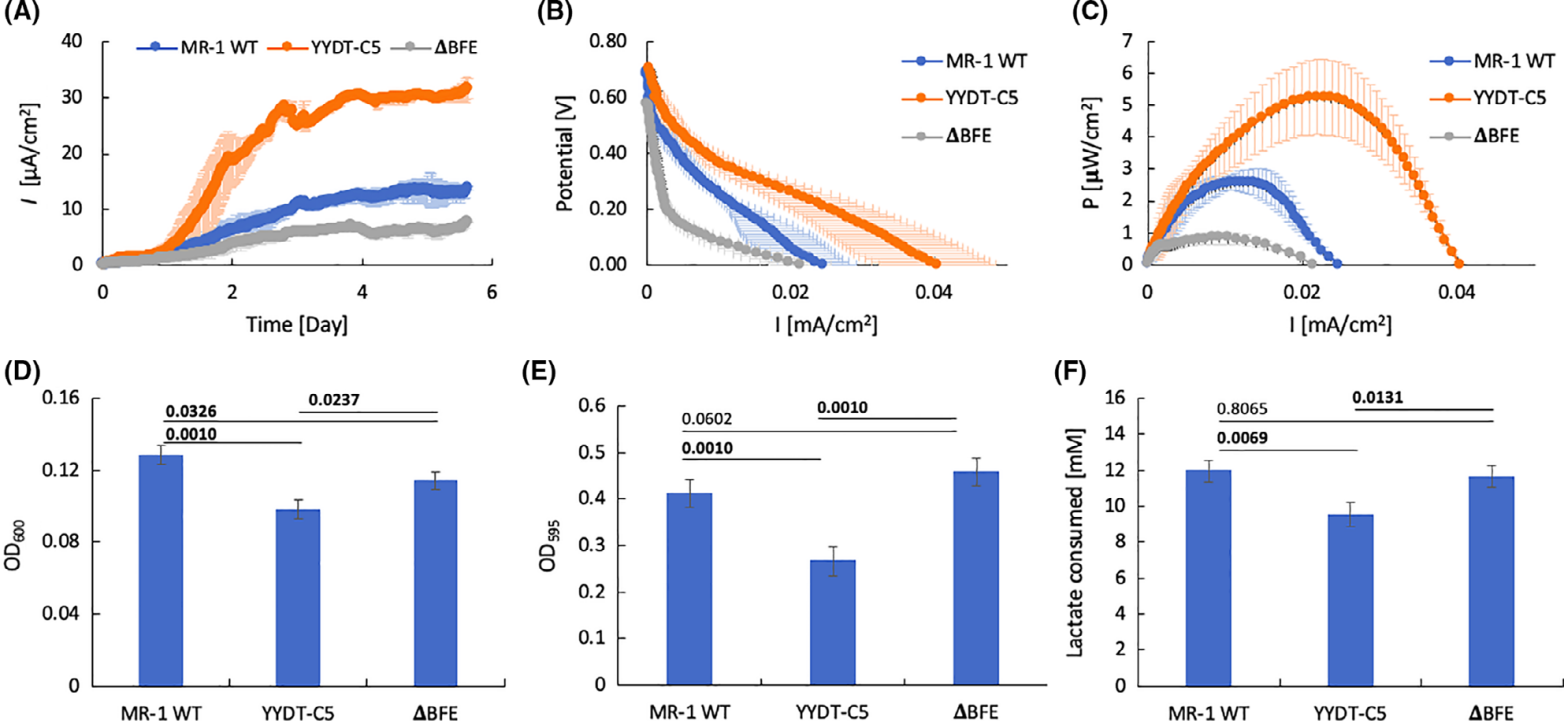
(A) Electric current density *I* versus elapsed time, (B) polarization curve (current density vs. potential) and (C) power density curve of MFC reactors with *S. oneidensis* MR‐1 WT (blue), MR‐1/YYDT‐C5 mutant (orange) and MR‐1 Δ*bfe* strains (grey) with 20 mM sodium lactate as sole carbon source. Measurements were conducted via Linear Sweep Voltammetry, as described above. Error bars represent the standard deviation of triplicate measurements. (D) Measured optical density at 600nm (OD_600_) of anodic culture of MFC reactors utilizing *S. oneidensis* MR‐1 WT, MR‐1/YYDT‐C5 mutant and MR‐1 Δ*bfe* strains with 20 mM sodium lactate as sole carbon source. Measurement was done using 1 cm cuvette (1 ml sample size). (E) Biofilm quantification using crystal violet assay: optical density at 595 nm (OD_595_) of cell‐bound crystal violet solution from anodic biofilm cells of the MFC reactors. F. The amount of lactate consumed by each reactor. Produced metabolites were mainly acetate, with succinate and pyruvate in trace amounts (data not shown). Measurements in Figure (D), (E) and (F) were done at the end of MFC experiment (day 13). Error bars represent standard deviation of triplicate measurements. *P* values on top of the bars denote differences between sample pairs based on nested mixed‐factor ANOVA test followed by Tukey’s HSD *post hoc* test. *P* values showing statistically significant (*P* < 0.05) differences are presented in bold.

The steady‐state bioelectrical current density generated by the MR‐1 WT reached 13.7 ± 0.3 µA cm^−2^, compared to 7.6 ± 0.1 µA cm^−2^ for the MR‐1 Δbfe and 31.5 ± 1.8 µA cm^−2^ for the MR‐1/YYDT‐C5 mutant (*P* < 0.05) (Fig. 3A; Table 2). After about 6 days of operation, MR‐1/YYDT‐C5 exhibited the highest bioelectrical current density vs. potential, compared to MR‐1 WT and MR‐1 Δbfe (Fig. 3B). The maximum output power density of the MR‐1 WT was 2.61 ± 0.35 µW cm^−2^, compared to 0.83 ± 0.19 µW cm^−2^ from the MR‐1 Δbfe, while MR‐1/YYDT‐C5 reached 5.25 ± 1.18 µW cm^−2^ (*P* < 0.05) (Fig. 3C and Table 2).

**Table 2:** The steady‐state bioelectrical current density and maximum output power density (power per unit electrode surface area) of the MFC running with MR‐1 wild‐type and mutants.

	Current density [µA cm^−2^]	Max. power density [µW cm^−2^]
MR‐1 WT	13.7 ± 0.3	2.61 ± 0.35
MR‐1 Δbfe	7.6 ± 0.1	0.83 ± 0.19
MR‐1/YYDT‐C5	31.5 ± 1.8	5.25 ± 1.18

The OD_600_ of anodic culture in the MR‐1 WT reactors reached 0.129 ± 0.005, while that of MR‐1/YYDT‐C5 and MR‐1 Δbfe peaked at a density of 0.098 ± 0.005 and 0.114 ± 0.005 respectively (Fig. 3D). The OD_595_ of cell‐bound crystal violet solution from anodic biofilm cells of the MR‐1 WT was found to be 0.411 ± 0.030, and 0.267 ± 0.031 for MR‐1/YYDT‐C5 and 0.458 ± 0.030 for MR‐1 Δbfe (Fig. 3E). This translated into the number of attached cells in the biofilm as (2.74 ± 0.18) × 10^5^ cm^−2^ for MR‐1 WT, (1.78 ± 0.24) × 10^5^ cm^−2^ for MR‐1/YYDT‐C5 and (3.05 ± 0.20) × 10^5^ cm^−2^ for MR‐1 Δbfe. Consumption of lactate in MR‐1 WT, MR‐1/YYDT‐C5 and MR‐1 Δbfe was measured to be 12.0 ± 0.6 mM, 9.5 ± 0.7 mM and 11.6 ± 0.6 mM respectively (Fig. 3F). The reduction of flavin by *bfe* gene knockout in MR‐1 Δbfe only produced 31% power, while the increase of flavin by overexpression of ribADEHC in MR‐1/YYDT‐C5 boosted power generation by twofold, in comparison with MR‐1 WT (Table 2). These results demonstrate that flavin‐enabled electron shuttling was the dominant mechanism of MFC‐based bioelectricity generation in *S. oneidensis* MR‐1, which is in‐agreement with previous study^18^. It also suggests that the introduction of gene cluster encoding flavin biosynthesis (e.g. pYYDT‐C5) into an established biofilm of MFCs has the potential to significantly enhance electricity production performance.

### Ultrasound‐mediated DNA delivery (UDD) to biofilms in microbial fuel cells (MFCs)

The transfer of pYYDT‐C5 plasmid into MR‐1 WT biofilms via UDD in an MFC was performed employing the set‐up shown in Fig. 1B, where the acoustic parameters and water bath properties are the same as in the previous study. The effect of pYYDT‐C5 on bioelectricity generation in established biofilms was investigated in a double‐compartment MFC set‐up. The MFC system with plasmid transfer via UDD (WT_P_US) was compared to several controls: a positive control with MR‐1/YYDT‐C5 strain (MR‐1/YYDT‐C5_US), and two negative controls: the addition of plasmid without ultrasound (WT_P) and ultrasound treatment without plasmid (WT_US).

**Fig. 4:**
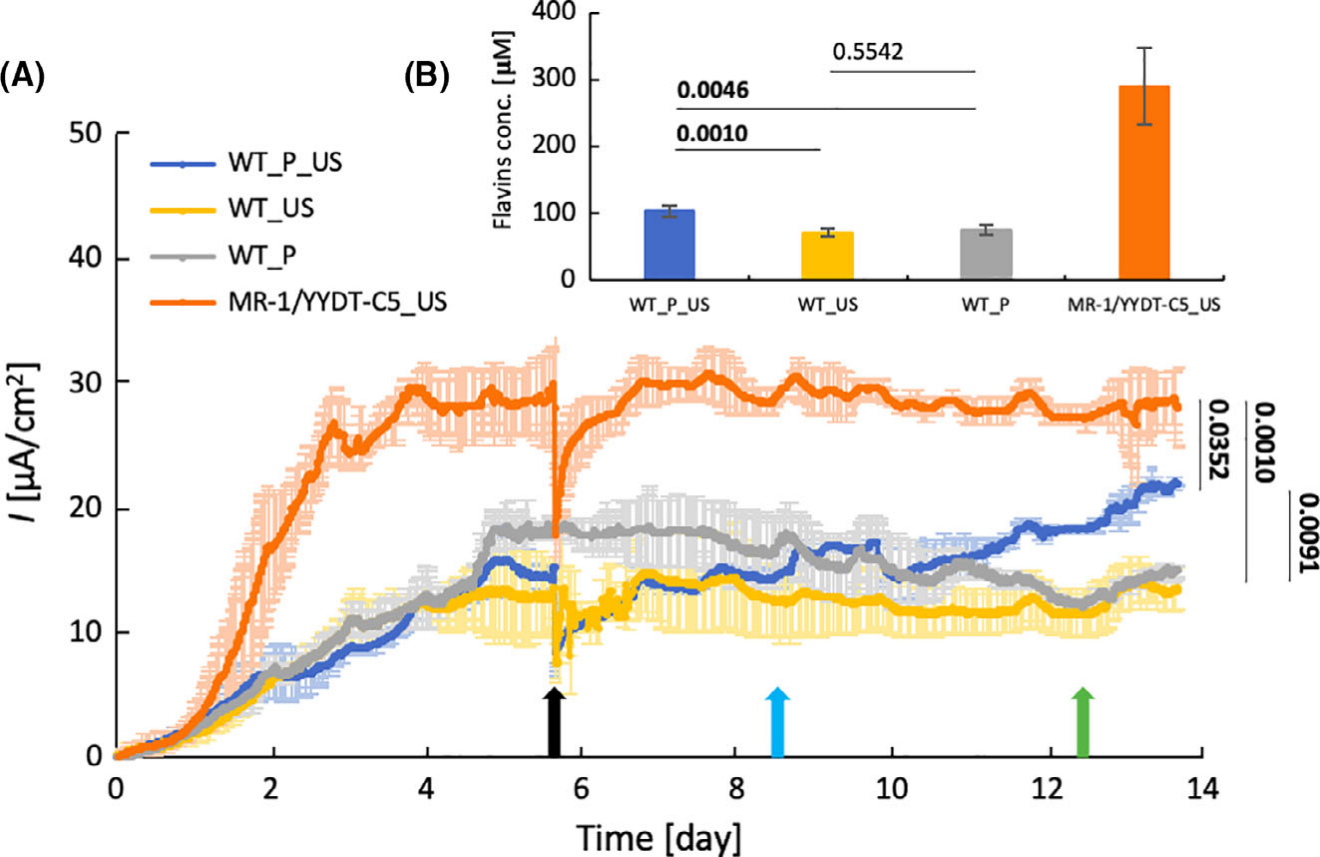
(A) Electric current density*I*of double‐compartment MFC reactors running at 1kΩ load with 20 mM initial concentration of sodium lactate; (B) extracellular flavins concentration of MFC reactors after 14 days of operation. Four different type of reactors: MR‐1/YYDT‐C5 strain (MR‐1/YYDT‐C5_US, orange), MR‐1 WT with addition of plasmid and ultrasound treatment (WT_P_US, blue), MR‐1 WT with only ultrasound treatment (WT_US, yellow) and WT with only addition of plasmid (WT_P, grey). Ultrasound was performed for 30s on day 6 (black arrow) for appropriate MFC set‐ups. On day 9, kanamycin (10 µg ml^‐1^) and 10 mM of additional lactate were added into each reactor (light blue arrow). On day 13, additional kanamycin was added to reach final concentration of 50 µg ml^‐1^ (green arrow). Shaded regions represent standard deviations of triplicate measurements. *P* values on top of the bars were calculated for the last day of measurement and denote differences between sample pairs based on nested mixed‐factor ANOVA test followed by Tukey’s HSD post hoc test. *P* values showing statistically significant (*P* < 0.05) differences are presented in bold.

Consistent with the previous experiment, the electricity generation of MR‐1/YYDT‐C5 positive control (28.0 ± 3.3 µA cm^−2^) was significantly higher than the WT systems throughout the experiment (Fig. 4A). Once the bioelectrical current generation reached steady state after approximately 4 days, the addition of plasmid and/or ultrasound treatment was conducted between day 5 and 6. Bioelectrical current production in all treated MFC systems dropped immediately after ultrasound treatment was performed, but it fully recovered after approximately 24 h (Fig. 4A). This observation indicates that ultrasound treatment can result in temporary disturbance of the MFC system, possibly due to the physical disruption of the biofilm structure by acoustic cavitation and/or mechanical stress. However, cells in the biofilm were able to restructure themselves and fully recover with no permanent detriment afterwards. It is important to note that the nature and extent of ultrasound physical effects depend critically on a number of acoustic parameters. These effects, which are primarily mechanical and thermal in nature, can serve to both disrupt biofilms and promote gene transfer. We believe that, for a given system, there will exist optimum sets of acoustic parameters and exposure protocols that minimize biofilm disruption and cell death while enhancing gene delivery. This subject lies beyond the scope of the current work and is a topic of ongoing study.

Forty‐eight hours after plasmid transfer using ultrasound treatment, the WT_P_US system started generating higher bioelectrical current than that of WT_US and WT_P. At the end of the experiment, the WT_P_US system generated a bioelectrical current of 21.9 ± 1.2 µA cm^−2^, 61% higher (*P* < 0.05) than that of the WT_US system (13.6 ± 1.6 µA cm^−2^) (Fig. 4A; Table 3). The application of UDD to treat the biofilms established within the MFC resulted in the increased production of flavins by the WT_P_US system over time. The WT_P system produced similar bioelectrical current to WT_US (14.9 ± 0.6 µA cm^−2^), indicating that bacterial transformation only occurred in treatments in which plasmids were introduced in the presence of ultrasound.

**Table 3:** Final electric current density (current per unit electrode surface area) and extracellular flavin concentrations of UDD‐treated MFC systems.

	Current density (µA cm^−2^)	Flavins concentrations (µM)
WT_P_US	21.9 ± 1.2	103.3 ± 8.3
WT_US (−ve control)	13.6 ± 1.6	70.9 ± 5.9
WT_P (−ve control)	14.9 ± 0.6	74.8 ± 7.3
MR‐1/YYDT‐C5_US (+ve control)	28.0 ± 3.3	289.7 ± 57.7

Three days after ultrasound treatment, kanamycin (10 µg ml^−1^) and lactate (10 mM) were added to all reactors to induce selection pressure for transformed cell growth and to maintain high electron donor concentration respectively (Fig. 4A, light blue arrow). A 3‐day time gap was selected to enable the transformed bacterial cells, which were maintained at room temperature, to produce the necessary proteins in low‐growth minimum media to resist the antibiotics. Additional kanamycin (40 µg ml^−1^) was added on day 13 (Fig. 4A, green arrow). The addition of antibiotics on the separate occasions had no detectable effect on the bioelectrical current produced by the controls, since the mode of action of kanamycin did not initiate immediate killing of cells but instead interferes with protein synthesis and prevents cell replication^16^. This indicated that the established cell density in those reactors had reached optimum concentration before the antibiotics was added and that the whole process was not catalyst‐limited. Injection of additional lactate on day 9 also had no detectable effect of improving performance on bioelectricity production, indicating that the reaction was not substrate‐limited either (Fig. 4A, light blue arrow).

The quantity of flavin electron shuttles secreted by the *Shewanella* strain played a significant role in influencing bioelectrical current generation in the MFC system (Yang *et al*., 2015). After 14 days of operation, the amount of extracellular flavins in each MFC reactor was quantified. The WT_P_US system produced an approximately 50% higher concentration (*P* < 0.05) of extracellular flavins (103.3 ± 8.3 µM) compared to the WT_US and WT_P systems (70.9 ± 5.9 µM, and 74.8 ± 7.3 µM respectively) (Fig. 4B, Table 3). Enhanced flavin production in the WT_P_US system was attributed to the additional synthesis pathway encoded in pYYDT‐C5 plasmid, which was introduced into the *S. oneidensis* biofilm via UDD. This quantitative analysis of flavin confirmed that the transfer of the plasmid was achieved via ultrasound. The MR‐1/YYDT‐C5 positive control system contained the greatest concentration of flavin (289.7 ± 57.7 µM), which is consistent with the bioelectrical current generation result.

The extraction and sequencing of plasmids from transformed cells in the WT_P_US MFC system (see the Supporting informations) provided additional evidence for the successful transfer of the pYYDT‐C5 plasmid into a *S. oneidensis* MFC biofilm. These results combined provide strong evidence of the ability of UDD to deliver desired genes *in situ* into bacterial biofilm. This demonstrates that UDD is able to enhance biofilm‐based bioelectrochemical performance in MFCs *in situ* without the need of restarting the bioreactor and re‐building the biofilm, which is highly desirable for large‐scale industrial applications involving continuous bioreactors.

## Discussion

### *In situ* plasmid uptake by bacterial cell in flow cell biofilms via UDD

To date, *in situ* bacterial transformations in biofilms have been limited to competent cells (Hendrickx *et al*., 2003). In this study, we attempted to non‐invasively and remotely introduce gene into non‐competent established biofilms *in situ* using ultrasound‐mediated DNA delivery (UDD). A pBBR1MCS‐2_P_Lux__sfGFP plasmid (8822 bp) was constructed using the broad‐host‐range cloning vector backbone pBBR1MCS‐2 and DNA fragments encoding *sfGFP* and the positive‐feedback *luxI* and *luxR* system (Prindle *et al*., 2012). We selected *sfGFP* and the positive‐feedback *luxI* and *luxR* system because they provide a strong green fluorescence signal in transformed bacteria cells (Scott *et al*., 2017). This pBBR1MCS‐2_P_Lux__sfGFP plasmid was employed as delivery DNA for *P. putida* UWC1 biofilms grown in a commercially available microfluidic flow cell (Sternberg and Tolker‐Nielsen, 2006) system (Fig. 1A). UWC1 was selected for UDD because it is an environmentally and industrially relevant bacterium and is not naturally competent (McClure *et al*., 1989).

We have shown that applying low‐frequency ultrasound (42 kHz) to UWC1 biofilms in the presence of pBBR1MCS‐2_P_Lux__sfGFP plasmid resulted in the *in situ* uptake of plasmid by bacterial cells, which expressed sfGFP within the biofilm after 5 h of incubation (Fig. 2). While it is technically difficult to directly measure the efficiency of bacterial transformation within the biofilm, it can be estimated by counting transformants over the total bacteria population in the effluent from the biofilm flow cell.

In this study, kanamycin was used as a selection pressure to enhance the impact of UDD on the general functionality of the biofilms, as seen from the increasing magnitude of green fluorescence signals over time (Fig. 2A, B, C and G). However, in industrial applications, different types of compounds beyond antibiotics can be used as selection pressure. The use of ‘selection pressure’ method could be particularly useful in applications such as industrial wastewater treatment, where the presence of contaminants can act as a form of selection pressure within bioreactors. For instance, copper is usually present in small amount in wastewater but sudden surges of copper content in wastewater may induce toxic shock in biofilms (Cabrero *et al*., 1998) and upset their bioreactors, leading to long term downtime and the release of untreated wastewater into the environment. Current mitigation methods to protect the environment and prevent government regulatory penalties include dilution to reduce copper concentration per unit volume of wastewater and/or procurement of specially formulated sludge to treat the high copper levels, incurring both financial costs and significant bioreactor downtime. The UDD technique described has the potential to bridge the gap, by transforming bacteria in the biofilm and sludge bioreactors to express appropriate genes (e.g. copper resistance) for the unexpected surge of toxic compounds in wastewater. The introduction of genes for biosynthesis of electron shuttles provided a benefit to the transformants in the case of MFC biofilm, the electricity production was enhanced as the consequence of the selection pressure (Fig. 4). Furthermore, in biodegradation and bioremediation application, the selection pressure can be the targeted organic contaminant itself, which can serve as the carbon source for the transformants containing specific genes to metabolize the organic contaminant.

We have demonstrated a novel UDD method for bacterial transformation within established biofilms growin in microfluidic flow cells. With this, bacterial cells within established biofilms acquired specific genes of interest (in this case, *luxI*, *luxR and sfGFP*) through bacterial transformation, enabling the biofilms to display new phenotypes and functionalities. Our previous work focused on bacteria transformation for cells in suspension (Song *et al*., 2007), and UDD has not been demonstrated in biofilms prior to this work. It has since been reported by others that UDD can be applied to Gram‐positive bacteria (Lin et al., 2010), but only for cells in suspension. The advantage of ultrasound for gene transfer over that of conventional transformation methods (electroporation, heat shock, conjugation, etc.) is that UDD can potentially be scaled‐up for industrial use. We have demonstrated successful bacteria transformation via UDD in biofilms growing in microfluidic cells of small volumes (i.e. 0.16 cm^3^). In addition to scalability, the UDD approach can be applied to the prevailing industrial systems whereas electroporation has to be employed in very specific optimized conditions that can only be generated in the laboratory. It will be interesting to examine whether bacterial transformation via UDD can be achieved in larger volume bioreactors and using transformation indicators beyond fluorescence signals.

### UDD induced in situ bacterial transformation in MFC

The double‐compartment MFC reactor employed in this study allowed reliable evaluation of the impact of UDD on bioelectricity output by MR‐1 strains exhibiting varying flavin production and bioelectricity generation capabilities. MR‐1 was selected as a model organism due to its unique extracellular electron transfer ability (Shi *et al*., 2007) and the fact that it is not naturally competent. The pYYDT‐C5 plasmid was chosen as delivery DNA into *S. oneidensis* MR‐1 WT biofilms as the plasmid contains the entire flavin biosynthesis gene cluster ribADEHC cloned from *Bacillus subtilis*, which has previously been shown to improve the bioelectricity generation of the transformed MR‐1 as compared to the MR‐1 WT (Yang *et al*., 2015).

We have demonstrated that applying low‐frequency ultrasound (42 kHz) to *S. oneidensis* biofilms growing on electrodes in the presence of plasmids results in the *in situ* uptake of pYYDT‐C5 plasmid by bacterial cells, which generated almost twice as much bioelectricity in the MFC after 8 days of incubation as compared to negative controls. The pYYDT‐C5 plasmid used here is a relatively large plasmid (10 450 Bp). While it is well recognized that transformation efficiency decreases with increasing plasmid size (Hanahan, 1983), our results showed that the UDD technology is not limited to the delivery of small plasmids, but is also effective for delivering relatively large plasmids as well. UDD is poorly understood, but it appears to be a physical phenomenon in which cell membrane permeability is transiently enhanced by cavitation activity (Song *et al*., 2007). By selecting the proper acoustic parameters (primarily frequency and acoustic pressure amplitude), it may even be possible to achieve bacterial transformation via UDD involving the uptake of mega plasmids and genomic DNA fragments (Taghavi *et al*., 1994).

UDD‐treated biofilms in MFC were only able to match around 70% of the level of bioelectricity generated by MR‐1/YYDT‐C5 positive control system by day 14 (Fig. 4A). Compared to the results for the application of UDD in flow cells biofilm, it is evident that bacterial transformation efficiency can be a limiting factor preventing treated biofilms from achieving the maximum theoretical productivity. To alleviate this limitation, the appropriate use of selection pressure can amplify the effects of UDD treatment on the biofilm to exhibit high productivity.

It has been previously suggested that the mechanism of transdermal protein delivery using low‐frequency ultrasound, such as 20 kHz, is attributed mainly to acoustic cavitation physical effects (Mitragotri *et al*., 1995; Tang *et al*., 2002; Prausnitz *et al*., 2004). It is possible that the mechanism of UDD in biofilms is similarly via acoustic cavitation where microbubbles, formed on the surface of or within biofilms, are made to volumetrically oscillate under periodic acoustic forcing. Associated with this behaviour is a host of physical effects that include fluid microstreaming, shock wave production, localized viscous heating and the formation of re‐entrant liquid jets due to asymmetric bubble collapse (Erriu *et al*., 2014; Vyas *et al*., 2019). Potential bioeffects on cells stemming from these physical effects of acoustic cavitation have been reviewed in the literature (Leighton, 1994; Coussios and Roy, 2008). For example, microstreaming breaks down boundary layers and promotes the convection of plasmids proximal to the cell surface. Jetting pokes small holes in the cell, leading to transient enhancement of cell membrane porosity. The biofilm matrix contains extracellular polymeric substances such as lipids, polypeptides and polysaccharides of diverse chemical charges (Wuertz *et al*., 1998; Wimpenny *et al.,* 2000; Flemming *et al*., 2007; Cao *et al*., 2011), and is an ideal adsorption material for extracellular DNA or plasmids to be introduced to the biofilms. The high cell density in the biofilms (Alpkvist *et al*., 2006), potentially coupled with proximity between the bacteria and plasmids of interest in the biofilm matrix, provides a suitable environment for ultrasound‐mediated horizontal gene transfer to take place within non‐competent bacterial biofilm communities. While ultrasound treatment and its associated physical effects can aid gene transformation in biofilm, the very same effects can also disrupt the biofilm structure and stability. At present, the mechanistic details of UDD remain elusive and further studies are required to optimize acoustic parameters and exposure protocol, such as ultrasound intensity, duration, duty cycle, types of transducer, and operating temperature and pressure, for more efficient and reproducible gene transfer.

### Scaling up UDD in biofilms for industrial applications

The goal of this study was to introduce new functionalities into non‐competent established biofilms in bioreactors of different scales via *in situ* UDD. Current conventional gene transfer techniques (such as electroporation, heat shock and conjugation) are optimized for small scale laboratory use (typically < 2 ml), but all have limitations that make them unsuitable for larger scale, *in situ* applications. Electroporation requires salt‐free conditions and typically kills > 90% cells with transformation usually limited to specific strains. Heat shock requires a rapid and drastic change in temperature (> 30°C) of the cells and their liquid medium. Conjugation requires direct physical contact between donor and recipient strains. Theoretically, ultrasound‐mediated DNA delivery (UDD) is capable of engineering bacterial culture and biofilms *in situ* without these inherent limitations at larger scale. In this study, UDD‐induced gene transfer on non‐competent biofilms grown in both microfluidic flow cells and microbial fuel cell (MFC) systems was successfully demonstrated, with working volumes of 0.16 cm^3^ and 300 cm^3^, respectively, achieving a significant scale‐up in operating volume using the same acoustic exposure system. To the best of our knowledge, there has not been any technique designed to enable bacterial transformation within biofilms *in situ* and/or in operating volumes larger than 2 ml.

These results provide solid evidence that UDD‐based techniques hold promise in terms of achieving efficient bacterial transformation at industrial scales. DNA fragments containing genes of interest may be introduced *in situ* into established biofilms cultured in bioreactors, reducing downtime and ensuring continuous operations. It may also be possible to influence gut microbiome of animals and human beings for agricultural or medical purposes, respectively, using this approach. Thus, the ability to alter the phenotype of established biofilms creates new possibilities for influencing their behaviour in environmental, industrial and medical settings. While UDD technology clearly has huge potential, further research into its physical mechanisms is required for optimization and/or industrial‐scale exploitation. Nonetheless, by demonstrating that *in situ* gene transfer in biofilms via UDD is possible for a bioelectrochemical system such as MFC, this exciting proof‐of‐concept opens the door to opportunities for the exploitation of this novel technology to enhance the controllability and efficiency of biofilm‐based processes in the environmental, industrial and medical contexts.

## Experimental procedures

### Chemicals, bacteria and plasmids

All chemicals were from Sigma‐Aldrich (United Kingdom) and used without modification unless otherwise stated. The strains and plasmids used in this study are listed in Table 1.

pBBR1MCS‐2_P_Lux__sfGFP plasmid (8822 bp), containing the broad‐host‐range cloning vector backbone pBBR1MCS2, *sfGFP* and the positive‐feedback *luxI* and *luxR* system, was employed as delivery DNA for *P. putida* UWC1 while pYYDT‐C5 (10 450 Bp, a gift from Prof Hao Song (Yang *et al*., 2015)), containing entire flavin biosynthesis gene cluster ribADEHC, was employed as delivery DNA for *S. oneidensis* MR‐1 WT. Briefly, plasmid DNA was extracted and purified from bacterial cultures at their respective mid‐exponential phase using a QIAprep Spin Miniprep kit (QIAGEN, Hilden, Germany). DNA concentration was determined using a NanoQuant Plate™ and Spark microplate reader (TECAN, Switzerland). More information on plasmid preparation can be found in Supporting informations.

### Construction of pBBR1MCS‐2_PLux_sfGFP plasmid

pTD103luxl_*sfGFP* (from Jeff Hasty, Addgene plasmid # 48885; http://n2t.net/addgene:48885; RRID:Addgene_48885) was cut via restriction digest using BglII then AvrII. The Plux_sfGFP fragment, containing *sfGFP* and the positive‐feedback *luxI* and *luxR* system, was isolated following separation via gel electrophoresis. P_lux__*sfGFP* was ligated into a pBBR1MCS‐2 plasmid backbone (from Kenneth Peterson, Addgene plasmid # 85168; http://n2t.net/addgene:85168; RRID:Addgene_85168) which had been linearized by restriction digest with BamHI and XbaI. The resulting pBBR1MCS‐2_P_lux__*sfGFP* plasmid was transformed into C2987 NEB‐5α *E. coli* which were screened via M13 colony PCR. Plasmids were extracted from positive transformants.

### Growth of *P. putida* UWC1 biofilms

Biofilms of *P. putida* UWC1 were grown in three‐channel flow cells (channel dimensions, 1 × 4 × 40 mm^3^; Merck, Germany) using 1/10th‐strength LB Lennox medium (Merck, Darmstadt, Germany) continuously supplied through a peristaltic pump. The flow system, consisting of 2 l glass bottles (Fisher Scientific, Loughborough, UK), Masterflex silicone tubing and peristaltic pump (Cole‐Palmer, United Kingdom), bubble trap and flowcells (Merck, Darmstadt, Germany), was assembled (Fig. S2) and sterilized as described previously (Sternberg and Tolker‐Nielsen, 2006). Each flow cell channel was inoculated with 0.3 ml overnight culture (diluted to an OD_600_ of 0.1) using a 1 ml syringe and 26G needle (BD, Franklin Lakes, NJ, USA). After inoculation, the medium flow was stopped for 1 h to allow initial attachment followed by continuous media flow with a flow rate of 10 ml h^−1^.

### Ultrasound apparatus

A standard 42‐kHz (±6%) ultrasonic cleaning bath (Model 3510E‐DTH; Branson Ultrasonics Corp., Danbury, CT, USA) with a maximum output power of 100 W was used in this study. The ultrasonic sound field was measured with a hydrophone (Type 8103; Bruel & Kjaer, Nærum, Denmark) and consisted of bursts with a modulation period of 10 ms and a modulation depth of about 90%. The sound field amplitude spectrum displayed a strong peak at 42 kHz with harmonics extending up to almost 500 kHz. These harmonics were all between −23 and −60 dB relative to the primary peak and could be have been caused by non‐linearity in the acoustic driver and cavitation noise in the water bath. The pulse‐average root‐mean‐square acoustic pressure was 170 kPa, corresponding to a pulse‐average acoustic intensity of 1.9 W cm^−2^. The mean and standard deviation of five measured waveforms was employed to estimate the peak‐positive acoustic pressure of the pulse, which was 398 ± 62 kPa and the measured peak‐negative acoustic pressure was 362 ± 41 kPa.

For each experiment, the bath was filled to the same level with type‐1 water at laboratory temperature (order 20°C) and local atmospheric pressure (order 1 bar). The dissolved air content was not controlled and was believed to vary from 85% to 95% of saturation. The biofilm sample holders used in each experiment (either a microfluidic flow cell or a microbial fuel cell) were submerged to the same depth and same lateral location within the bath. No exogenous cavitation‐promoting particles, drops or microbubbles (*i.e*. cavitation nuclei) were employed in the study.

### Ultrasound DNA delivery into flow cell biofilms

Four sets of flow cells were used to cultivate biofilms in which 3‐day‐old biofilms were treated using 4 sets of exposure conditions: with both addition of plasmid and ultrasound treatment (+P/+U), with only ultrasound treatment (−P/+U), with only addition of plasmid (+P/−U) and without both addition of plasmid and ultrasound treatment (−P/−U). The peristaltic pump connected to the flow cells was switched off prior to ultrasound treatment. 0.3 ml of 10 mM CaCl_2_ solution with or without 1 µg ml^−1^ of pBBR1MCS‐2_P_lux__sfGFP plasmid (coding for green fluorescence protein) was injected into the appropriate flow cells. Tubing at both ends of the flow cells was clamped, and the flow cells were incubated at room temperature for 10 min. The flow cells were fully submerged in the ultrasonic cleaning bath (described above) and appropriate flow cells were subjected to ultrasound treatment for 10 s. After resting for a further 10 min, the clamps at both ends of the flow cells were removed and the peristaltic pump was switched back on at a flow rate of 10 ml h^−1^. After 2 h of flow, the growth media were changed to 1/10th‐strength LB medium containing 10 µg ml^−1^ kanamycin for the rest of the experiment and waste bottles were replaced as and when required. Biofilms samples within the flow cells were viewed using ZEISS LSM 900 with Airyscan 2 confocal laser‐scanning microscope (Carl Zeiss AG, Oberkochen, Germany) for signs of green fluorescence signal.

Bacterial samples were collected from within the flow cells using sterile needles and syringes, resuspended in sterile 0.9% NaCl solution, and spread on LB agar plates containing 50 µg ml^−1^ kanamycin. Colonies formed on the agar plates were resuspended in sterile 0.9% NaCl solution and underwent plasmid extraction procedure using Monarch® Plasmid Miniprep Kit (New England Biolabs, Ipswich, MA, USA) according to manufacturer’s instructions. Concentration of plasmid samples was determined using a NanoQuant Plate™ and Spark microplate reader (TECAN, Switzerland), while size of plasmids in samples was compared with pBBR1MCS‐2_PLux_sfGFP using horizontal gel electrophoresis systems (Bio‐Rad, Hercules, CA, USA) according to manufacturer’s instructions.

The intensity amplitude of the ultrasound field was determined in the plasmid transfer system as previously described (Song *et al*., 2007).

### MFC reactor set‐up

Dual‐compartment MFC reactors with a working volume of 300 ml per compartment were used to investigate bioelectrical current production. The anode was made of 3.0 × 3.0 cm^2^ carbon cloth (H23, 95 g m^−2^; Quintech, Gloucestershire, UK). The cathode was carbon cloth containing a Pt catalyst (1 mg cm^−2^, PtC 60%, 2.5 × 4.0 cm^2^; FuelCellStore). Titanium wire was used to connect the electrodes to the outside of the reactors. Nafion© 117 was used as the exchange membrane to separate the two compartments.

Reactors were assembled and initially filled with deionized water, then autoclaved to achieve sterility. The water was then replaced with appropriate media; standard M9 minimal salt, supplemented with trace minerals, amino acids and vitamins, was chosen as the anodic compartment media and prepared according to Cao *et al*. (2011) with slight modifications. The list of chemicals and their corresponding concentrations in each stock are given in Tables S1–S3 The M9 salt solution was autoclaved before the trace elements were added in 1:100 dilution from their stocks via 20 µm pore‐size membrane sterile filtration. The final medium was supplemented with 20 mM sodium DL‐lactate and 0.75 mM IPTG as pYYDT‐C5 plasmid inducer. Cathodic compartment media was phosphate buffer saline (PBS), prepared by dissolving two 500 mg PBS tablets in 1 l deionized water, then autoclaved to achieve sterility.

Fixed resistors of 1 kΩ were used to complete the circuit. Keithley Instrument Datalogger 2701 was used to measure the voltage across the resistor every 10 min. Before bacterial injection, the anodic compartment was bubbled with nitrogen for 15 min to create anaerobic condition. Throughout the experiment, the anodic and cathodic compartments were continuously gassed with nitrogen and air respectively. Three independent replicate reactors were run for each different system.

### Polarization and power density curve construction

The power production of wild‐type *S. oneidensis* MR‐1 and its flavin deficient/enhancement mutant counterparts was measured via polarization curve construction. A potentiostat (PalmSens 4‐channel Multi EmStat^3+^) was used to perform linear sweep voltammetry (LSV) on the MFC reactors, with the voltage varied between the theoretical open‐circuit potential to zero. (E_begin_ = 0.8V, E_end_ = 0.0V, E_step_ = 0.1V, scan rate = 0.1 mV s^−1^). The power density curve was then constructed using values derived from multiplying the applied voltage and the corresponding measured bioelectrical current, yielding the total electric power in accordance with Ohm’s law:

(1) P=IV.

Here, *P* is total electrical power, *I* is bioelectrical current, and *V* is the applied voltage. The total power measured in this manner was then normalized by the anode surface area, yielding the power density.

### Planktonic and biofilm cell quantification

The concentration of planktonic cells in the reactor was determined by its optical density (more commonly known as optical absorbance) using a light spectrometer (UV‐1800 Shimadzu) to measure light absorption at a wavelength of 600 nm. A cuvette length of 1 cm with sample size of 1 ml was employed, with fresh anodic media as a blank to exclude background reading.

Biofilm cell concentration was measured using a crystal violet assay. The anode was immersed in 20 ml of 0.1% crystal violet solution and then washed twice with 20 ml sterile deionized water. Finally, the cell‐bound crystal violet was dissolved in 20 ml of 70% isopropanol. The absorbance at 595 nm of four independent 100 µl replicates of the final solution was measured and normalized with background reading of crystal violet originating from a cell‐free anode. The OD_595_ value is proportional to the number of cells attached on the biofilm, with the OD‐to‐cell number conversion was calculated using standard curve of known cell density.

### Metabolites quantification

The amount of remaining lactate and produced metabolites within the reactors were quantified via high‐performance liquid chromatography (HPLC) equipped with acid column Hi Plex – H (250 × 4.6 mm, particle size 8 µm; Agilent, Santa Clara, CA, USA). The eluent was 0.005 M H_2_SO_4_ with flow rate of 0.6 ml min^−1^, and signal was detected using UV detector at 210 nm and 55°C. One ml of reactor medium was sampled and filtered using a 0.2 μm membrane filter to remove cells before being measured for its chemical concentration. Prior to the MFC experiment, standard curves of lactate and possible metabolites (acetate, pyruvate, format and succinate) were constructed.

### *In situ* plasmid transfer into *S. oneidensis* MR‐1 in MFC

The effect of pYYDT‐C5 plasmid transfer into *S. oneidensis* MR‐1 via ultrasound was investigated in terms of the bioelectrical current production in an MFC system. Late‐stationary phase culture of MR‐1 was injected into the reactor to achieve an initial OD of 0.01. After reaching stable bioelectrical current generation across 1 k± Ω resistor, 0.1 µg ml^−1^ of the plasmid was injected into appropriate reactors (*WT_P_US)*. Ultrasound was then performed for 30s at a frequency 42 kHz (±6%) to transfer the plasmid into the cell, and bioelectrical current production was monitored. As controls, reactors with wild‐type (*WT_US*) and MR‐1/YYDT‐C5 strain (*MR‐1/YYDT‐C5_US*) without further addition of plasmid were also experimented as controls. Another control of WT strain with plasmid addition, but without ultrasound treatment, was also measured to exclude the effect of such treatment (*WT_P*). Three independent replicate reactors were run for each system. Injection of kanamycin and lactate was done using sterile syringe and needle through one of the ports on the side of the reactor. Kanamycin was added from 50 mg ml^−1^ stock to achieve the desired final concentration in the reactor. Lactate was added from its 1 M stock, pre‐filter sterilized to achieve sterility.

### Flavin quantification

Fluorescence spectroscopy was used to detect and quantify riboflavin and flavin mononucleotide (FMN) secreted by *S. oneidensis* in the MFC reactor. 100 μl of the cell‐free supernatant of anodic media was transferred to a clear 96‐well plate and read at 440 nm excitation and 525 nm emission. Four independent replicate aliquots were run for each reactor, and the background fluorescence was corrected by using fresh anodic media as the blank. Flavin concentration was determined using standard curves previously constructed with known concentrations of FMN (concentration range: 1 to 1 ng ml^‐1^).

### Plasmid sequencing and verification

At the end of MFC experiment, the anodic biofilm was collected and centrifuged to obtain cell pellets. Plasmid extraction protocol using a Monarch® Plasmid Miniprep Kit was performed and the obtained plasmid was quantified using NanoDrop and a plate reader. The primers *PRTac‐SF3_for* and *ribC‐02_R8_rev* (Supporting informations) were used to sequence and identify the necessary plasmid fragment to confirm successful transfer of pYYDT‐C5 plasmid into *S. oneidensis*.

### Statistical analysis

For all measurements involving replication, nested mixed‐factor ANOVA tests followed by Tukey’s HSD post hoc tests were performed to determine the significance between different treatment groups. A *P* value of < 0.05 denotes a statistically significant difference between the conditions of interest.

## Funding Information

W.E.H. acknowledges support from EPSRC (EP/M002403/1 and EP/N009746/1). C. K. Ng acknowledges financial support from Commonwealth Scholarship Commission in the form of Commonwealth Rutherford Fellowship (SGRF‐2017‐471). S. L. Putra acknowledges financial support from Jardine Foundation in the form of Jardine‐Oxford Postgraduate Scholarship.

## Conflict of interest

The authors claim no conflict of interest.

## Supporting information


**Fig. S1**. The growth media outputs of the microbial flowcells under various conditions: presence of plasmids with ultrasound treatment (+P/+U), presence of plasmid without ultrasound treatment (+P/−U), absence of plasmid with ultrasound treatment (−P/+U), and absence of both plasmid and ultrasound treatment (−P/−U).
**Fig. S2**. The flow system used to culture biofilms consisting of growth media bottles, silicone tubing, peristaltic pump, bubble trap, flowcells, waste bottles and an ultrasound water bath.
**Fig. S3**. The plasmid map of pBBR1MCS‐2_plux_sfGFP.
**Table S1**. Ingredients of vitamin stock (×100).
**Table S2**. Ingredients of mineral stock (×100).
**Table S3**. Ingredients of amino acid stock (×100).
