# Supplementary material for: Genetic engineering biofilms in situ using ultrasound‐mediated DNA delivery
Source: Microb Biotechnol. 2021 May 16;14(4):1580–93. doi: 10.1111/1751-7915.13823 (PMC8313276; doi:10.1111/1751-7915.13823)
Supplement: Supplementary file 1 — Fig. S1. The growth media outputs of the microbial flowcells under various conditions: presence of plasmids with ultrasound treatment (+P/+U), presence of plasmid without ultrasound treatment (+P/−U), absence of plasmid with ultrasound treatment (−P/+U), and absence of both plasmid and ultrasound treatment (−P/−U). Fig. S2. The flow system used to culture biofilms consisting of growth media bottles, silicone tubing, peristaltic pump, bubble trap, flowcells, waste bottles and an ultrasound water bath. Fig. S3. The plasmid map of pBBR1MCS‐2_plux_sfGFP. Table S1. Ingredients of vitamin stock (×100). Table S2. Ingredients of mineral stock (×100). Table S3. Ingredients of amino acid stock (×100). [file MBT2-14-1580-s001.pdf]

Supplementary Materials

Genetic engineering biofilms *in-situ* using ultrasound-mediated DNA delivery

Chun Kiat Ng[$], Samuel Putra[$], Joseph Kennerley, Robert Habgood, Ronald A. Roy, Jason L. Raymond, Ian P. Thompson* and Wei E. Huang*

Department of Engineering Science, University of Oxford, Parks Road, OX1 3PJ, Oxford, UK.

$ The authors contribute equally to the manuscript.

[*]Corresponding author: Department of Engineering Science, University of Oxford, Parks Road, OX1 3PJ, Oxford, United Kingdom.

Email wei.huang@eng.ox.ac.uk and ian.thompson@eng.ox.ac.uk

Tel: +44 1865 283786 and +44 1865 283789.

Supplementary Figure

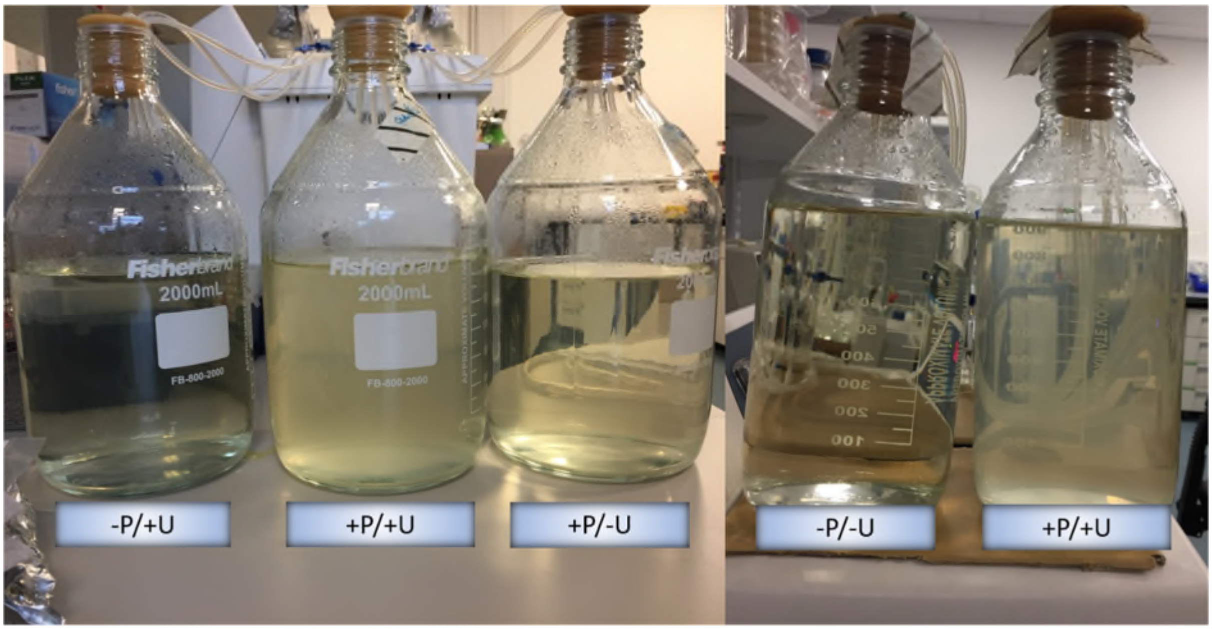

Figure S1. The growth media outputs of the microbial flowcells under various conditions: presence of plasmids with ultrasound treatment (+P/+U), presence of plasmid without ultrasound treatment (+P/-U), absence of plasmid with ultrasound treatment (-P/+U), and absence of both plasmid and ultrasound treatment (-P/-U).

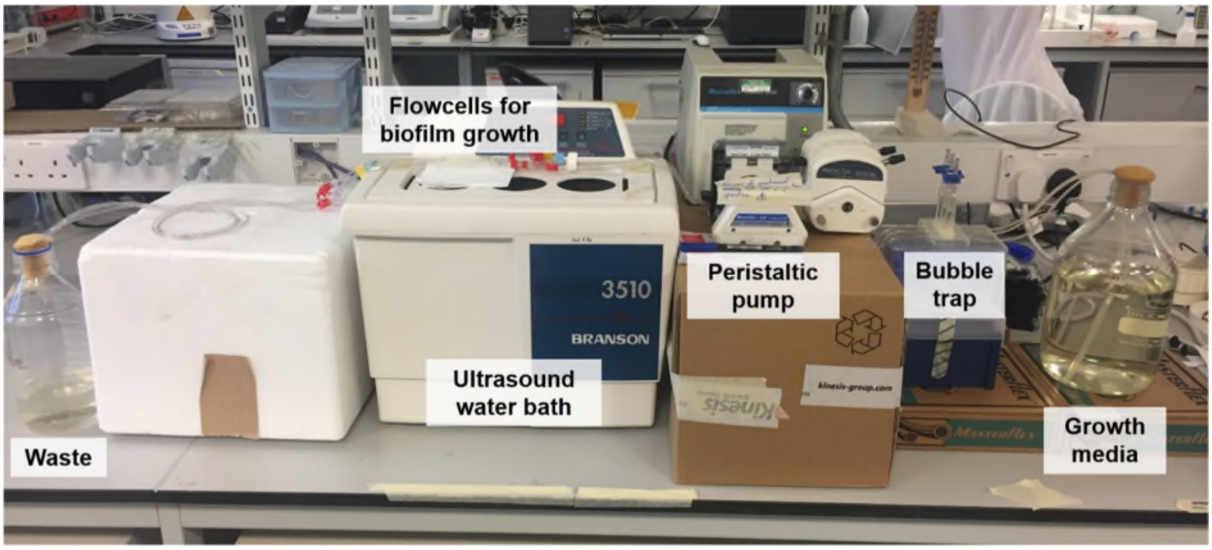

Figure S2. The flow system used to culture biofilms consisting of growth media bottles, silicone tubing, peristaltic pump, bubble trap, flowcells, waste bottles and an ultrasound water bath.

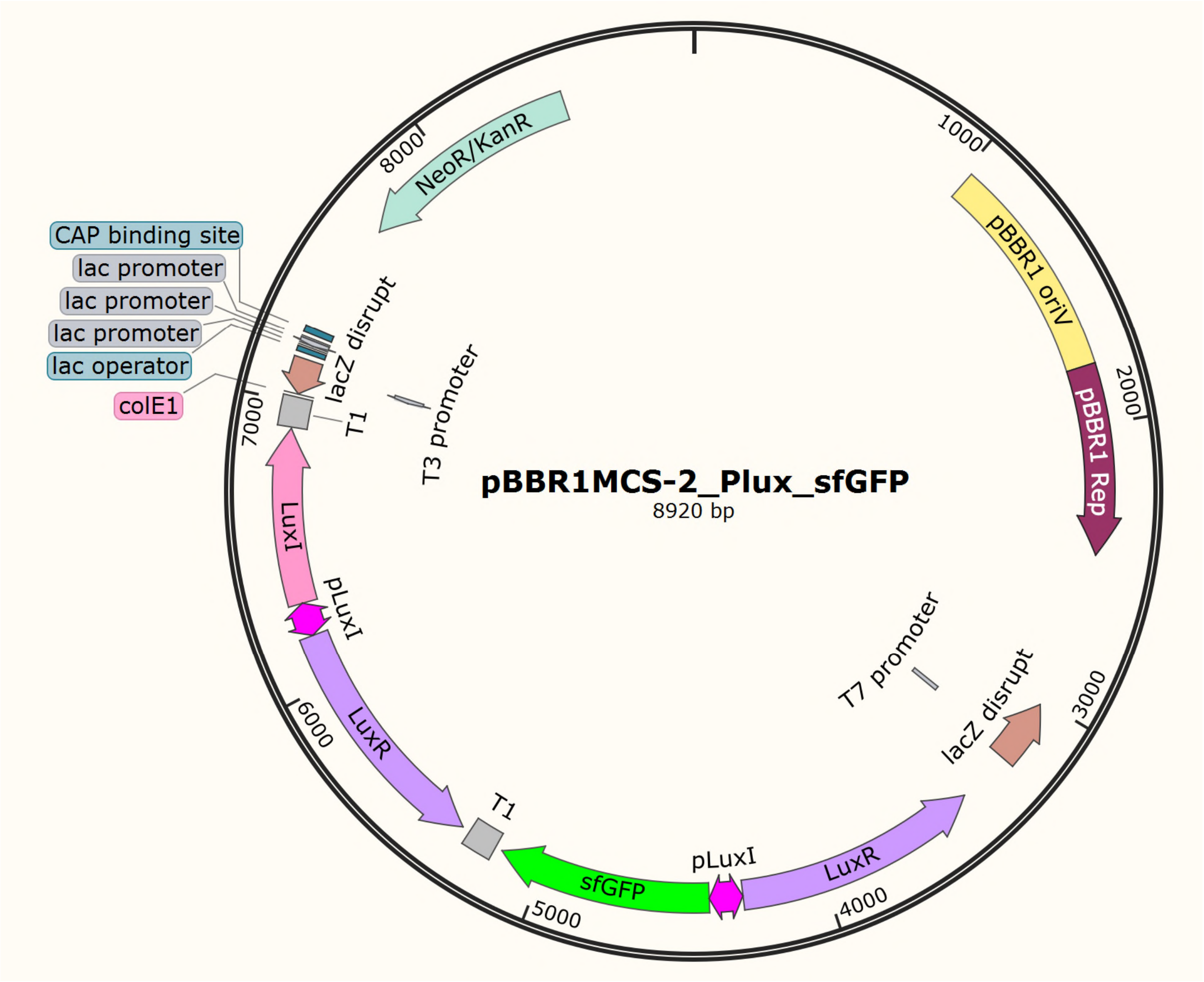

Figure S3. The plasmid map of pBBR1MCS-2_plux_sfGFP.

Supplementary Table

Table S1: Ingredients of vitamin stock (x100)

Chemical	FW	mg/L
Biotin (d-biotin)	244.3	2
Folic acid	441.1	2
Pyridoxine HCl	205.6	10
Riboflavin	376.4	5
Thiamine HCl 1.0 H_2O	355.3	5
Nicotinic acid	123.1	5
d-Pantothenic acid, hemicalcium salt	238.3	5
B12	1355.4	0.1
p-Aminobenzoic acid	137.13	5
Thioctic acid (or lipoic acid)	206.3	5

Table S2: Ingredients of mineral stock (x100)

Chemical	FW	g/L
Nitrilotriacetic acid	199.1	1.5
$MgSO_4.7H_2O$	246.48	3
$MnSO_4.H_2O$	169.02	0.5
NaCl	58.44	1
$FeSO_4.7H_2O$	277.91	0.1
$CaCl_2.2H_2O$	146.99	0.1
$CoCl_2.6H_2O$	237.93	0.1
$ZnCl_2$	136.28	0.13
$CuSO_4.5H_2O$	249.68	0.01
$AlK(SO_4)_2.12H_2O$	474.38	0.01
H_3BO_3	61.83	0.01
$Na_2MoO_4.2H_2O$	241.95	0.025
$NiCl_2.6H_2O$	237.6	0.024
$Na_2WO_4.2H_2O$	329.86	0.025

Table S3: Ingredients of amino acid stock (x100)

**Chemical**	**FW**	**g/L**
L-Glutamic acid	147.13	2
L-arginine	174.2	2
DL-serine	105.09	2

Sequencing results

pYYDT-C5 fragment to be detected by *PRTac-SF3_for* and *ribC-02_R8_rev* for UDD confirmation in MFC

Forward:

NNNNGGNNNNNNNNNAGAGGAGAATCTAGTATGTTCCACCCAATCGAAGAAGCTTTAGATGCTTTA
AAAAAAGGTGAAGTTATCATCGTTGTTGATGATGAAGATCGTGAAAACGAAGGTGATTTCGTTGCTT
TAGCTGAACACGCTACTCCAGAAGTTATCAACTTCATGGCTACTCACGGTCGTGGTTTAATCTGTACT
CCATTATCTGAAGAAATCGCTGATCGTTTAGATTTACACCCAATGGTTGAACACAACACTGATTCTCA
CCACACTGCTTTCACTGTTTCTATCGATCACCGTGAAACTAAAACTGGTATCTCTGCTCAAGAACGTT
CTTTCACTGTTCAAGCTTTATTAGATTCTAAATCTGTTCCATCTGATTTCCAACGTCCAGGTCACATCTT
CCCATTAATCGCTAAAAAAGGTGGTGTTTTAAAACGTGCTGGTCACACTGAAGCTGCTGTTGATTTA
GCTGAAGCTTGTGGTTCTCCAGGTGCTGGTGTTATCTGTGAAATCATGAACGAAGATGGTACTATGG
CTCGTGTTCCAGAATTAATCGAAATCGCTAAAAAACACCAATTAAAAATGATCACTATCAAAGATTTA
ATCCAATACCGTTACAACTTAACTACTTTAGTTGAACGTGAAGTTGATATCACTTTACCAACTGATTTC
GGTACTTTCAAAGTTTACGGTTACACTAACGAAGTTGATGGTAAAGAACACGTTGCTTTCGTTATGG
GTGATGTTCCATTCGGTGAANAACCAGTTTTAGTTCGTGTTCNNTCTGAATGTTTAACTGGTGATGTT
TTCGGTTCTCANCGTTGTGATTGTGGTCCACAATTACNCGCTGCTTTAAACCAAATCGCTGCTGAAG
GTCGNGGNGTTTNNTAAACTTACGTCANNNAGGTCNNNGTATCGGTTTAATCANNAAATTAAAAG
CTTANAAATTANNNNAACAAGGTTANAANNNNGNTNNNNCTANNNNNNNNTNNNNNNNNNN
NNNNNNNNNNNNNNNNNNNNNNNNNNNNNNNNNNNNNNNNNNNNNNNNNNNNNNNNNANNNN
NNNNNNNNNNNNNNNNNNCNNNNANNNNNNNNNNNNTANNNNNNNNNNNNNNNNNNNNNNN
NNNNNNNNNNNNNNNNNNNNNNNNNNNAA

Reverse:

NNNNNGCTTCNNNGTNNNCCNTTTTCNNNTTGTTCAGTTAATTCTTTTGATACCGTTGAATTTACGT
TCAGAACGGATACGTTTGTACCATTCGATTTTGATAGCAGCACCGTAAACTTCTTTGGTTGAAATCGA
ATAAGTTAACTTCGATAGATGGTTGTTTCTGGACGTTTTTCGTAGAAAGTTGGTTTGTAACCGATGTT
ACAAACACCGTTTGTAAACTTCACCGTTAACTTCAGCTTTAACAGCGTAAACACCAGTTGGTGGAAC
GATGTAAGAGTTGTTTAAACCAACGTTAGCAGTTGGGAAACCGATAGTACGACCACGTTTATCACCG
TGGATAACGATACCTTTGATGAAGTATGGTTGACCTAATAAAACGTTAGCTAATTCAACATCACCGTT
TTGTAAAGCAGTACGGATGTAAGAAGAAGAGATTTTTTTATCTTGTTCAGTTAATTTTTCAACCATAG
TACAACCAGCTTTACCATCTAAATCATCTGGCATAGTTTTCATAGTACCTTTACCGTATTTACCGTAAG
TGAAATCGAAACCAGCAACAGCGTGTTGAACGTTTAAACCGATGATGTATTGATCGATGAATTGTTT
TGGAGATAAAGAAGCGAAAACTTCGTTGAATTTAACAACGTATAAAACTTCAGTACCTAATTGTTCG
ATTTGGTTGATTTTATCTTCTAATGGAGTGATTAAATCTTTTGGTTCTTTATCACGACCTAAAACGTGA
GATGGGTGTGGGTGGAAAGTCATAACAGCTAAAGTTAAACCTTTTTCTTCAGCGATTTGTTTAGCAG
TACCGATAACTTTTTGGTGACCTAAGTGAANNCCATCGAAGTAACCTAAAGCCNNAACAGATTTAGC
TTGNTCTCNTTGATAATGGNGGGGNNNNNNNNNNGGGGAAANTNTNNNCNNNAGTTNNNNNCN
NNNNNNNNNNNNTANNNNAAANAACGGTTANNTNNNNNNTNNNNNNNNNNNNNNNTTGACTA
NNNNACANATNNNNNNN

Sequencing result of UDD-treated MFC-biofilm plasmid

Forward:

NNNNGGGNNNNGAAGAGGAGAATCTAGTATGTTCCACCCAATCGAAGAAGCTTTAGATGCTTTAA
AAAAAGGTGAAGTTATCATCGTTGTTGATGATGAAGATCGTGAAAACGAAGGTGATTTCGTTGCTTT
AGCTGAACACGCTACTCCAGAAGTTATCAACTTCATGGCTACTCACGGTCGTGGTTTAATCTGTACTC
CATTATCTGAAGAAATCGCTGATCGTTTAGATTTACACCCAATGGTTGAACACAACACTGATTCTCAC
CACACTGCTTTCACTGTTTCTATCGATCACCGTGAAACTAAAACTGGTATCTCTGCTCAAGAACGTTCT
TTCACTGTTCAAGCTTTATTAGATTCTAAATCTGTTCCATCTGATTTCCAACGTCCAGGTCACATCTTCC
CATTAATCGCTAAAAAAGGTGGTGTTTTAAAACGTGCTGGTCACACTGAAGCTGCTGTTGATTTAGC
TGAAGCTTGTGGTTCTCCAGGTGCTGGTGTTATCTGTGAAATCATGAACGAAGATGGTACTATGGCT
CGTGTTCCAGAATTAATCGAAATCGCTAAAAAACACCAATTAAAAATGATCACTATCAAAGATTTAAT
CCAATACCGTTACAACTTAACTACTTTAGTTGAACGTGAAGTTGATATCACTTTACCAACTGATTTCG
GTACTTTCAAAGTTTACGGTTACACTAACGAAGTTGATGGTAAAGAACACGTTGCTTTCGTTATGGG
TGATGTTCCATTCGGTGAANAACCAGTTTTAGTTCGNGTTCACTCTGAATGTTTAACTGNNGATGTTT
TCGGTTCTNACCGTTGTGATTGTGGTCCACAATTACNCGNTGCTTTAAACCAAATCGCTGCTGAAGG
TCGNNNTNTTTTATNANACTTACGTCANNNAGGTNNNGGTNTCGGTTNAATCAACAAATTAAAAGC
TTACAATTACANNNACAAGGTTANAAANNNNNNNNNNTAANNANNNNNNNNNNNNNNNNNNN
NNNNNNNNNNNNNNNNNNNNNNNNNNNNNNNNNNNNNNNNNNNNNNNNNNNNNNNNNNNN
ANNNNNNNCNNNNNNNNNNNNNNANNNNNNNNNNNNNNNNNNNNNNNNNNNNNNNNNNNN
NNA

Reverse:

NNNNNCTTCTTTGTTTATCTTTTTCGATTTGTTCAGTTAATTCTTTGATACCGTTGAATTTACGTTCAG
AACGGATACGTTTGTACCATTCGATTTTGATAGCAGCACCGTAAACTTCTTGGTTGAAATCGAATAA
GTTAACTTCGATAGATGGTTGTTCTGGACGTTTTTCGTAGAAAGTTGGTTTGTAACCGATGTTACAAA
CACCGTTGTAAACTTCACCGTTAACTTCAGCTTTAACAGCGTAAACACCAGTTGGTGGAACGATGTA
AGAGTTGTTTAAACCAACGTTAGCAGTTGGGAAACCGATAGTACGACCACGTTTATCACCGTGGATA
ACGATACCTTTGATGAAGTATGGTTGACCTAATAAAACGTTAGCTAATTCAACATCACCGTTTTGTAA
AGCAGTACGGATGTAAGAAGAAGAGATTTTTTTATCTTGTTCAGTTAATTTTTCAACCATAGTACAAC
CAGCTTTACCATCTAAATCATCTGGCATAGTTTTCATAGTACCTTTACCGTATTTACCGTAAGTGAAAT
CGAAACCAGCAACAGCGTGTTGAACGTTTAAACCGATGATGTATTGATCGATGAATTGTTTTGGAGA
TAAAGAAGCGAAAACTTCGTTGAATTTAACAACGTATAAAACTTCAGTACCTAATTGTTCGATTTGGT
TGATTTTATCTTCTAATGGAGTGATTAAATCTTTTGGTTCTTTATCACGACCTAAAACGTGAGATGGG
GTGTGGGGTGGAAAGTCATAACAGCTAAAGTTAAACCTTTTTCTTCAGCGATTTGTTTAGCAGTACC
GATAACTTTTTGGTGACCTAAGTGAACACCATCGAAGTAACCTAAAGCNATAACNNNTTTAGNTTGN
TCTTCTTTGATTAANNGNGGGGGTGAATGANNNNNAAAANTTT
